# The Logistics of Medication and Patient Flow in Video-Based Virtual Clinics During a Sudden COVID-19 Outbreak in Taiwan: Observational Study

**DOI:** 10.2196/37880

**Published:** 2022-06-10

**Authors:** Ying-Hsien Chen, Hui-Wen Wu, Ching-Chang Huang, Jen-Kuang Lee, Li-Tan Yang, Tse-Pin Hsu, Chi-Sheng Hung, Yi-Lwun Ho

**Affiliations:** 1 Department of Internal Medicine National Taiwan University Hospital Taipei Taiwan; 2 National Taiwan University Hospital Telehealth Center National Taiwan University Hospital Taipei Taiwan

**Keywords:** COVID-19, telemedicine, video-based virtual clinic

## Abstract

**Background:**

The COVID-19 pandemic was well controlled in Taiwan until an outbreak in May 2021. Telemedicine was rapidly implemented to avoid further patient exposure and to unload the already burdened medical system.

**Objective:**

To understand the effect of COVID-19 on the implementation of video-based virtual clinic visits during this outbreak, we analyzed the logistics of prescribing medications and patient flow for such virtual visits at a tertiary medical center.

**Methods:**

We retrospectively collected information on video-based virtual clinic visits and face-to-face outpatient visits from May 1 to August 31, 2021, from the administrative database at National Taiwan University Hospital. The number of daily new confirmed COVID-19 cases in Taiwan was obtained from an open resource.

**Results:**

There were 782 virtual clinic visits during these 3 months, mostly for the departments of internal medicine, neurology, and surgery. The 3 most common categories of medications prescribed were cardiovascular, diabetic, and gastrointestinal, of which cardiovascular medications comprised around one-third of all medications prescribed during virtual clinic visits. The number of virtual clinic visits was significantly correlated with the number of daily new confirmed COVID-19 cases, with approximately a 20-day delay (correlation coefficient 0.735; *P*<.001). The patient waiting time for video-based virtual clinic visits was significantly shorter compared with face-to-face clinic visits during the same period (median 3, IQR 2-6 min vs median 20, IQR 9-42 min; rank sum *P*<.001). Although the time saved was appreciated by the patients, online payment with direct delivery of medications without the need to visit a hospital was still their major concern.

**Conclusions:**

Our data showed that video-based virtual clinics can be implemented rapidly after a COVID-19 outbreak. The virtual clinics were efficient, as demonstrated by the significantly reduced waiting time. However, there are still some barriers to the large-scale implementation of video-based virtual clinics. Better preparation is required to improve performance in possible future large outbreaks.

## Introduction

COVID-19, caused by SARS-CoV-2, rapidly escalated to a worldwide pandemic from late 2019 to early 2020 [1,2]. More than 150 million confirmed cases had been reported worldwide at the end of April 2021. Social distancing, mask wearing, and even lockdowns have been used worldwide to try and control the pandemic. Vaccination for SARS-CoV-2 was first approved in December 2020, and by the end of April 2021, 25 million doses of vaccine had been administered worldwide [3]. To continue medical care and minimize unnecessary contact, many countries have implemented virtual clinics for patients with chronic conditions during the pandemic [4-11]. The platform and workflow of such virtual clinics continues to evolve to match the demands of medical needs during different phases of the pandemic [12].

In Taiwan, thanks to the timely use of case-based and population-based interventions, including border control, enhanced surveillance, contact tracing, travel restrictions, and quarantine, the number of confirmed COVID-19 cases has been well controlled, with only 1129 cases reported at the end of April 2021 [13,14]. However, a community outbreak occurred in early May 2021 that resulted in a rapid increase in the number of cases from 1100 to over 15,000 in less than 3 months. The Taiwan government raised the nationwide epidemic alert level from II to III on May 15, 2021. The vaccination rate was also suboptimal at that time. The first AstraZeneca vaccine was administered on March 3, 2021, and at the end of April 2021, fewer than 60,000 individuals had received at least one dose of vaccine.

During the outbreak, most patients with COVID-19 in Taiwan were hospitalized for isolation, monitoring, and treatment. One consequence of the rapid increase in COVID-19 hospitalizations was that medical resources were directed to pandemic control as opposed to regular medical work. For example, around 1100 negative-pressure isolation rooms around Taiwan were directly controlled by the government [15]. From January to March 2020, the number of medical visits fell by 6.8% compared to the same period in 2019 [16]. Meanwhile, emerging data showed that the virus could be spread by patients who were asymptomatic and that viral RNA can be detected in nasal and throat swabs up to 11 days after contact in a patient who is asymptomatic [17]. Therefore, regular outpatient visits by patients with chronic diseases decreased substantially. To deal with this situation, the Taiwan government reduced restrictions on telehealth to allow physicians to speak to patients with stable chronic conditions via virtual clinics, either by video teleconferencing or telephone call. The Bureau of National Health Insurance (NHI) in Taiwan also started to reimburse for virtual clinic visits from May 15, 2021, during the pandemic [18]. Regular refilling of prescriptions for this group of patients was also allowed during a virtual clinic visit. Virtual clinics were then gradually initiated nationwide, from community clinics to medical centers. This process was carefully controlled over 3 months [19]. The use of virtual clinics also decreased gradually 3 months after the outbreak. This is the first time that reimbursement for a virtual clinic has been granted in Taiwan.

The aim of this study was to present the logistics of medication and patient flow for video-based virtual clinics during the May 2021 outbreak in Taiwan at a tertiary hospital. Although there have been some previous reports on the use of virtual clinics during the COVID-19 pandemic worldwide [4-11], our study reports the implementation of virtual clinics in a country with few confirmed cases and good infection control for 16 months from January 2020. We analyzed video-based virtual clinic visits from May to August 2021 at National Taiwan University Hospital (NTUH) in Taipei. We also discuss potential improvements that can be made in preparation for the next outbreak, as well as opportunities to further develop the use of telehealth in the future.

## Methods

### Ethics

This study was approved by the Institutional Review Board of National Taiwan University Hospital (Taipei, Taiwan; 202202037RINC).

### Study Design

We designed this retrospective study to evaluate video-based virtual clinic visits during the May 2021 COVID-19 outbreak in NTUH. The numbers of patients with chronic conditions who used the video-based virtual clinic and who attended face-to-face clinic visits were obtained from our electronic health database. *International Classification of Diseases, Tenth Revision, Clinical Modification* (*ICD-10-CM*) codes were used to extract the diagnoses from the database. The *ICD-10-CM* codes used in this study are as follows: diabetes mellitus (E08, E09, E10, E11, E13), hypertension (I10, I15), coronary arterial disease (I20, I21, I22, I23, I24, I25), hypertensive heart disease without heart failure (I11), atrial fibrillation (I48), enlarged prostate with lower urinary tract symptoms (N40.1), hyperlipidemia (E78), Parkinson disease (G20), and congestive heart failure (I50). The characteristics of patients who attended the video-based virtual clinic were also analyzed. Furthermore, associations between the number of daily confirmed COVID-19 cases and the numbers of patients who visited our virtual or face-to-face clinics were also analyzed. The care of patients who were hospitalized with COVID-19 in Taiwan is directed and coordinated by the Taiwan Centers for Disease Control, based on location and the availability of negative-pressure rooms and trained personnel. As the number of cases at a single hospital may not reflect the severity of the outbreak, we used the total number of cases collected at all places around Taiwan to reflect the situation.

### Video-Based Virtual Clinic Implementation at Our Institute

#### Regulations

Regulations on the implementation of telemedicine were revised in Taiwan in 2018. Hospitals or clinics are only permitted to set up virtual clinics when following special regulations. However, the ability to prescribe medications via virtual clinics is not routinely granted and can only be performed in a few emergency situations. NTUH, a tertiary teaching hospital in Taipei, has provided a video-based virtual clinic service after obtaining approval in 2019, before the COVID-19 pandemic. The software, platform, and hardware for the implementation of video-based virtual clinics was therefore well established. However, the service fee for video-based virtual clinic appointments was not covered by the NHI in Taiwan before the COVID-19 pandemic. After the May 2021 COVID-19 outbreak, the Bureau of NHI agreed to cover the fee for the virtual clinics (telephone or video-based) during the pandemic. The prescription of medications during virtual clinic visits was also allowed.

#### Payment

Reimbursement by the NHI for virtual clinic visits during the pandemic was confined to patients with stable chronic illnesses, such as hypertension, diabetes, and heart failure with a stable condition. Before the pandemic, these patients received long-term follow-up at our hospital every 3 months with medication prescriptions.

#### Appointment

**Figure 1:**
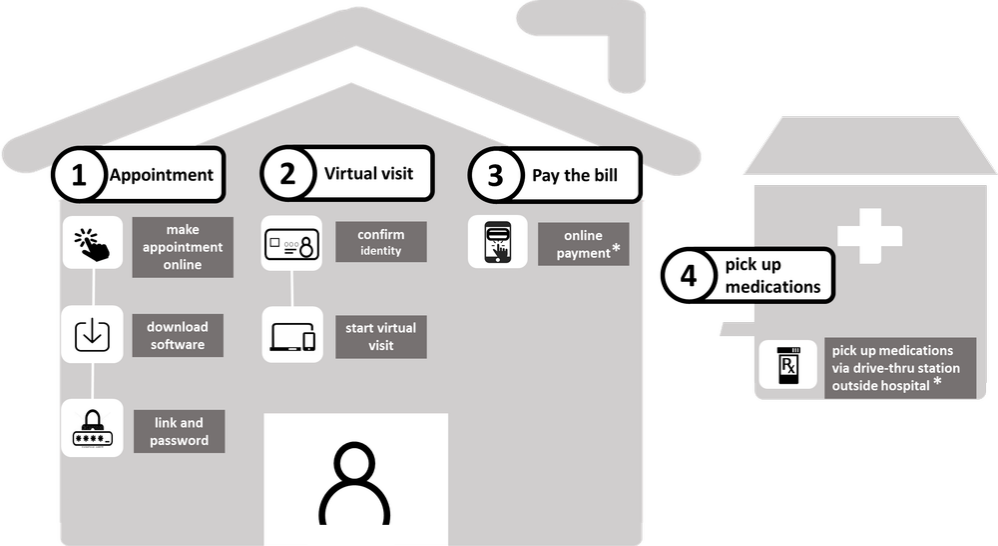
The workflow of our video-based virtual visit. (1) Appointment: eligible patients could make an appointment online and download the necessary software in advance. (2) Virtual visit: patients enter the virtual waiting room before the appointment time. Patients show their National Health Insurance card to the camera to confirm their identity. The physician then starts the virtual visit. (3) Pay the bill: patients or caregivers can pay the bill online (*or inside the hospital, according to their choice). (4) Medication pick-up: patients or caregivers pick up their medications via a drive-through station served by a pharmacist outside the hospital (*or inside the hospital, according to their choice).

Patients who agreed to use video-based virtual clinics could make an appointment online with the telehealth center in NTUH. Webex software, a teleconference link, use instructions, and a temporary username and password were then automatically emailed to the patient a day before their appointment. The workflow of the virtual clinic system is shown in Figure 1.

#### Video-Based Clinic Visit

Patients entered the virtual waiting room before their virtual visit. To confirm their identity, a video-based clinic visit was used instead of a telephone call. Patients provided their NHI card (mostly with a photo) at the beginning of the video conference to confirm their identity. The physicians in our telehealth center then interviewed the patient via videoconference with full-HD quality in an isolated clinic to maximize privacy. A record of the interview was documented in our health information system during the visit by the physician.

#### Medication Logistics

After the video-based clinic visit, the patients or caregivers should come to the hospital to collect their prescribed medications and pay the bill. The prescribed medications were reviewed by the pharmacist after a video-based clinic visit. A rapid medication collection station that was served by a pharmacist was set up outside the hospital to minimize the exposure of those visiting. The patients or caregivers collected the prescribed medications from the station outside the hospital, in a drive-through–like service, or inside the hospital according to their choice. An online bill-paying service was provided by the hospital from around July 2021. Patients could choose to pay online or inside the hospital according to their choice. The number of pills of the prescribed medications were obtained from our electronic health database, and the medications were classified into major clinical categories.

#### Waiting Time and Visit Time

We calculated the time used during the virtual clinic visits by our patients. The patient waiting time for a virtual clinic visit was defined as the time from when they entered the virtual waiting room to the initiation of the physician visit. These time points were recorded by the Webex software and retrieved from the electronic health database, retrospectively. The physician visit time was defined as the time between the initiation and the end of the physician visit. We also calculated the time used during face-to-face clinic visits during the same time period and across the same 9 specialties at our hospital during the study period. The waiting time for face-to-face clinic visits was defined as the time from when the patient registered with their NHI card after arriving for the physician visit, as recorded in our electronic medical record system. The physician visit time for face-to-face clinic visits was defined as the time between the initiation and the end of the physician visit.

### Statistical Analysis

Descriptive statistics were used in this study to analyze the data. Continuous variables are expressed as mean and SD. Cross-correlation was used to illustrate the association between the number of daily confirmed COVID-19 cases in Taiwan, and daily face-to-face clinic visits and daily virtual clinic visits at our hospital. The patient waiting time and physician waiting time in our video-based clinic were compared with those in face-to-face clinics using the Wilcoxon rank sum test. A *P* value of .05 or less was considered to indicate a statistically significant difference.

## Results

A total of 782 patients used the video-based virtual clinic between May and August 2021. Their mean age was 63.1 (SD 17.9) years, and 302 (38.6%) were male. The mean number of diagnoses for each patient was 3.8 (SD 2). Nine clinical departments in our hospital were involved in the video-based virtual clinic project (Table 1). The diagnosis listed was not mutually exclusive.

The medications prescribed in the virtual clinic are shown in Table 2. The internal medicine department had the highest number of virtual clinic visits, followed by the neurology and surgery departments. The medications most commonly prescribed in the virtual clinic were cardiovascular, diabetic, and gastrointestinal medications, of which cardiovascular medications comprised around one-third of all medications prescribed during this period.

The daily average number of virtual clinic visits was 13.5 (IQR 10.3-16.7) during the study period. During the same period, an average of 1886 (IQR 1750-2022) patients with chronic conditions visited our face-to-face clinic daily for long-term prescriptions. The number of face-to-face clinic visits was 2455 in the same period in 2020.

**Figure 2:**
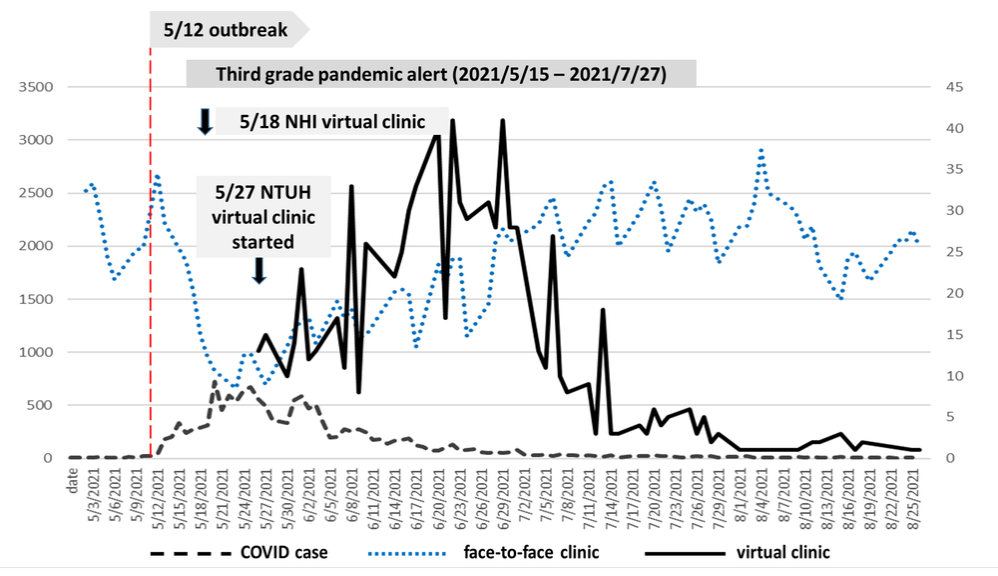
Time curve of daily new COVID-19 cases (gray dashed line), NTUH face-to-face clinic visits (blue dotted line), and NTUH virtual clinic visits (black line; the scale is shown on the secondary y-axis on the right side) between May 1 and August 31, 2021. After the community outbreak around May 12, the number of face-to-face clinic visits decreased immediately, while the number of virtual clinic visits increased much later, with a 20-day delay. The Central Epidemic Command Center announced a level 3 pandemic alert from May 15 to July 27, 2021. NHI: National Health Insurance; NTUH: National Taiwan University Hospital.

Figure 2 demonstrates the association between the number of virtual clinic patients (black line), number of face-to-face clinic patients (blue dotted line), and the number of new daily COVID-19 cases (black dashed line) in Taiwan. The outbreak began on May 12, plateaued in mid-May to early June 2021, and then subsided gradually in August 2021. The Central Epidemic Command Center raised the nationwide pandemic alert from level 2 to level 3 on May 15, 2021, and finally downgraded it to level 2 on July 27, 2021.

**Table 1:** Baseline characteristics of the patients visiting the virtual clinic between May and August 2021.

Demographic			Patients (N=782)
Sex (male), n (%)			302 (38.6)
Age (years), mean (SD)			63.1 (17.9)
**Department of virtual clinic, n (%)**			
	Internal medicine	438 (56.0)	
	Neurology	89 (11.4)	
	Surgery	68 (8.7)	
	Family medicine	47 (6.0)	
	Psychiatry	43 (5.5)	
	Ophthalmology	40 (5.1)	
	Obstetrics and gynecology	31 (4.0)	
	Urology	23 (2.9)	
	Orthopedics	3 (0.4)	
**Most common diagnosis, n (%)**			
	Diabetes mellitus	40 (5.1)	
	Hypertension	40 (5.1)	
	Coronary arterial disease	38 (4.9)	
	Hypertensive heart disease without heart failure	24 (3.1)	
	Atrial fibrillation	15 (1.9)	
	Enlarged prostate with lower urinary tract symptoms	15 (1.9)	
	Hyperlipidemia	13 (1.7)	
	Parkinson disease	13 (1.7)	
	Congestive heart failure	11 (1.4)	

**Table 2:** Medications prescribed during video-based virtual clinic visits.

Category	Pill prescribed (N=123,745), n (%)
Cardiovascular	35,453 (31.5)
α-Blocker	924 (0.8)
β-Blocker	6242 (5.6)
Vasodilator	280 (0.2)
ACEI^a^	154 (0.1)
ARB^b^	7336 (6.5)
Calcium channel blocker	6749 (6.0)
Antiadrenergic agent	168 (0.1)
Antiarrhythmia	3018 (2.7)
Digoxin	68 (0.1)
Diuretic	2552 (2.3)
Lipid-lowering agent	6408 (5.7)
Other cardiovascular	1554 (1.4)
Hematologic	10,202 (9.1)
Endocrine and metabolic	17,466 (15.5)
Antihistamine	979 (0.9)
Respiratory	1917 (1.7)
Gastrointestinal	14,017 (12.5)
Immunosuppressive	684 (0.6)
Anti-infectious	1352 (1.2)
Urologic	2044 (1.8)
Dermatological	54 (0.0)
Ophthalmological	150 (0.1)
Combination pill	76 (0.1)
Insulin pen needle	1092 (1.0)
Nutritional	2806 (2.5)

^a^ACEI: angiotensin-converting enzyme inhibitor.

^b^ARB: angiotensin II receptor blocker.

The number of face-to-face clinic visits at our hospital decreased immediately after the outbreak. However, there was a delay of 20 days before the number of virtual clinic visits increased. The cross-correlation between the number of face-to-face clinic visits and new daily COVID-19 cases was significant and without a lag (correlation coefficient –0.79; *P*<.001), while the cross-correlation between the number of virtual clinic visits and new daily COVID-19 cases was significant with a lag of 20 days (correlation coefficient 0.735; *P*<.001).

**Figure 3:**
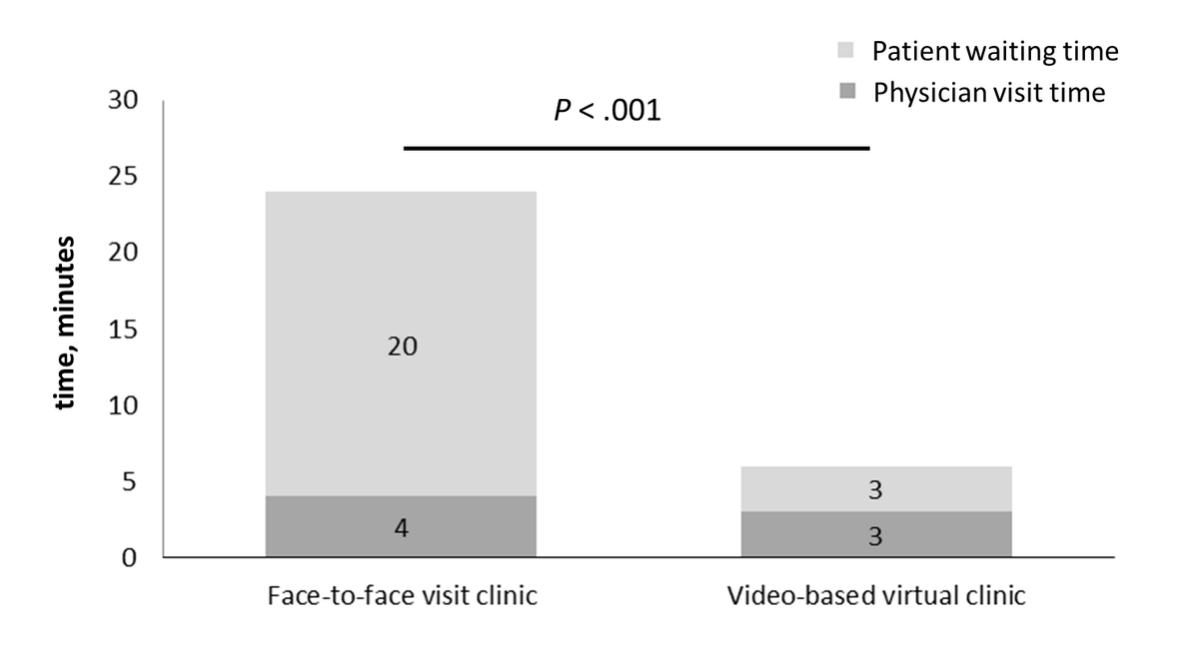
The patient waiting times and physician visit times for video-based virtual clinics and face-to-face clinic visits. The median patient waiting time was 20 (IQR 9-42) minutes for face-to-face clinic visits and 3 (IQR 2-6) minutes for video-based virtual clinic visits (rank sum *P*<.001). The median physician visit time was 4 (IQR 2-9) minutes for face-to-face clinic visits and 3 (IQR 2-5) minutes for video-based virtual clinic visits (rank sum *P*<.001).

The median patient waiting time for the video-based virtual clinic was 3 (IQR 2-6) minutes. However, among the specialties with a higher virtual visit volume (over 80 during this period), the waiting time was significantly less compared with specialties that had a lower volume (rank sum *P*<.05). The median physician visit time was 3 (IQR 2-5) minutes. We also collected the waiting and visit duration times during the same study period and the same 9 specialties for the face-to-face clinic visits (n=99,806). The median waiting time for the face-to-face clinic was 20 (IQR 9-42) minutes, while the median visit time was 4 (IQR 2-9) minutes. The patient waiting time for video-based virtual clinic visits was significantly shorter (median 3 min vs 20 min; *P*<.001, rank sum test) compared with that for face-to-face clinic visits, while the physician visit time was also significantly shorter for video-based virtual clinic visits (median 3 min vs 4 min; *P*<.001, rank sum test) (Figure 3). This reduction in patient waiting time was significant across all 9 specialties (all *P*<.05, rank sum test).

## Discussion

### Principal Findings

Our retrospective analysis revealed several major findings: video-based virtual clinics can be implemented rapidly after the outbreak for patients with chronic illnesses who require regular follow-up, the number of video-based virtual clinic visits at our hospital increased with a 20-day delay after the COVID-19 outbreak and was correlated with the number of daily new COVID-19 cases, and video-based virtual clinics shortened the patient waiting time significantly compared with face-to-face clinic visits. The implementation of video-based virtual clinics during the outbreak decreased unnecessary exposure of patients with chronic illnesses, who are a relatively vulnerable group. The time and cost of travel to face-to-face clinic visits can also be saved.

The implementation of virtual clinics during the COVID-19 pandemic has been reported in different countries and in different disease specialties [4-11]. The scale, rate, and barriers for the implementation of virtual clinics in different countries may not be the same. Gilbert et al [9] reported on the experience of virtual clinic implementation in a UK tertiary orthopedic center during the COVID-19 pandemic. A COVID-19 action team was established in their hospital to implement the virtual clinic visits, which were conducted via a telephone call or a videoconference. Considerable administrative, clinical, and technical resources were directed to the virtual clinic, and over 90% of the visits could be completed via the virtual clinic 1 month after its implementation. The majority of virtual clinic visits were performed via the telephone (telephone vs video, around 9 vs 1). In feedback from the patients after a virtual clinic visit, 94% using the telephone service expressed that they would like to use the virtual clinic next time, compared with only 44% of those using the videoconference. This report showed the experience of scaling up the amount of virtual clinic appointments during a severe and prolonged outbreak. However, the barriers for the implementation of videoconferencing as the method for virtual clinic visits warrant further studies.

Kim et al [11] also reported the results of teleconsultation, which was temporarily allowed in Korea during the COVID-19 pandemic. The researchers obtained teleconsultation data from the NHI claims database. From February to June 2020, a total of 567,390 cases received teleconsultation, of which 46.4% were claimed by clinics. Teleconsultations accounted for only 0.25% of the total medical use during this period. The most common major disease category for these consultations was circulatory diseases, followed by endocrine, nutritional, and metabolic diseases. From the patient number versus time curve, the increase in the number of virtual clinic visits in tertiary and teaching hospitals was delayed compared with the immediate increase in the number of appointments at primary health clinics. After the COVID-19 situation had become stable, the number of teleconsultations in hospitals decreased, but those in primary health clinics persisted. The researchers also noted that teleconsultations were provided most often to 3 types of patients: (1) those scheduled for follow-up visits, (2) those with chronic illnesses such as diabetes and hypertension, and (3) those living in high-infection areas. These findings are consistent with our results. However, the COVID-19 outbreak was more severe in Korea compared with Taiwan, which may partially explain the differences observed in the number of teleconsultations used between Korea and Taiwan.

In our study, there was a 20-day lag cross-correlation between the number of virtual clinic visits and the number of daily new COVID-19 cases. Several factors, including demand and supply, may have contributed to this phenomenon. With regards to supply, hospitals needed time to adjust to the workflow and implement the virtual clinics. The information and technology team had to be activated to redesign the processes, including appointment booking, physician-patient interviews, electronic medical records, billing, and pharmacy. With regards to demand, the patients with chronic illnesses could have obtained medications from pharmacies or primary care clinics. They may also have waited for the outbreak to subside. Hence, they may have postponed the regular follow-up visits. From our data, it is possible that the use of virtual clinics at our hospital during the outbreak was mainly driven by the severity of the outbreak. After the number of daily new COVID-19 cases decreased, the use of the virtual clinics also decreased, even though NHI reimbursement persisted.

Although our findings suggest that it is possible to implement video-based virtual clinics for the care of patients with chronic illnesses during the pandemic, the number of video-based virtual clinic visits was still low compared with the number of face-to-face clinic visits. Part of the delay in the increase in video-based virtual clinic visits may be because most patients were waiting for a rapid decrease in the daily number of new COVID-19 cases. However, other potential barriers to prevent the prompt conversion to and uptake of virtual clinics still exist [20,21]. A guide to overcoming these barriers and to facilitate the implementation of virtual clinics has been proposed [22]. Here, we discuss some of the potential barriers observed during the May 2021 outbreak at a tertiary medical center in Taiwan.

The service fee for the virtual clinic visits was reimbursed after the May 2021 outbreak. The registration fee (for the appointment) and copayment for medications from virtual clinic visits are the same as those for face-to-face clinic visits. The NHI payment therefore largely reduced the economic barrier. However, the patients needed to have equipment and access to the internet to be able to use the virtual clinics. Patients in extreme economic conditions may therefore not have been able to afford a virtual clinic visit.

To confirm the identity of a patient for a virtual clinic visit, we only allowed videoconferencing and not telephone calls. Videoconferencing has the advantage of the patient being able to confirm their identity. Furthermore, through teleconference software, the patient also has the autonomy to enter the virtual waiting room before their appointment time. In contrast, patients can simply wait for a call from the clinic if using the telephone as the modality of a virtual visit. Our data showed a shorter waiting time compared with conventional face-to-face visits. A retrospective study also showed that video visits were associated with fewer 90-day emergency department visits or hospitalizations compared with telephone visits as the modality of telemedicine for patients with heart failure, after adjusting for multiple predictors [23].

However, video-based virtual clinics require a minimum internet speed and camera resolution. Moreover, patients with chronic illnesses are mostly older adults, who may have lower levels of digital literacy. The use of teleconference equipment is not always straightforward for this patient group. Patients with hearing or cognitive dysfunction also have more difficulties in using these technologies [24]. The nurses in our telehealth center had to spend a lot of time instructing patients on how to use the software to perform the videoconference. Moreover, family members of the patients frequently had to ask for leave to stay at home and operate the video software and hardware to complete the virtual clinic visit. Video-based virtual clinics may also raise concerns regarding the security of patient information.

Performing a physical examination during a telephone-based virtual visit is almost impossible; however, it is still difficult during a video-based virtual clinic visit. A patient-assisted clinical examination guide has been proposed to help physicians evaluate physical signs during a videoconference [25]. However, only inspection and vital sign measurements can be reliably performed in a routine video-based virtual clinic visit. Novel technologies such as an electronic stethoscope, pulse oximeter, or wearable electrocardiography can only be used in certain conditions. This barrier may preclude the use of virtual clinic visits for the initial visit of a new patient [26].

Regulations in Taiwan require that a pharmacist must deliver medications directly to patients and explain their use to avoid medication error [27]. During the COVID-19 pandemic, this regulation has been upheld. Therefore, after a virtual clinic visit, patients or caregivers still had to go to the hospital to collect prescriptions and medications. In Taiwan, several hospitals including ours provide a drive-through collection method to help minimize contact between patients and pharmacists when collecting their medications. Novel pharmacy and drug delivery services are urgently needed in the near future to maximize the benefits of telehealth care during the COVID-19 pandemic [28,29].

There were several limitations to this study. First, the retrospective observational design of the study precludes any causal inference. Second, the data were collected at a tertiary medical center, which has accommodated many confirmed cases of COVID-19. The use of virtual clinics in a community hospital and primary care clinic cannot be determined from our data. Further analysis of the use of virtual clinics in other institutes is warranted to better understand the resource use and to facilitate better planning for future outbreaks. Finally, the study period was only 3 months, so the effects of current virtual clinic implementation on health outcomes over a longer time period are still not clear.

### Conclusion

Our study demonstrated the implementation of video-based virtual clinics during the COVID-19 pandemic at a tertiary teaching hospital in Taiwan. Video-based virtual clinics minimized contact between patients and health workers, and saved the patients’ time both in waiting time and travel to the hospital. Video-based virtual clinics can be rapidly adopted at a medical center to provide continuous care during disease outbreaks. An increase in the number of confirmed COVID-19 cases heralded a surge in the use of the virtual clinic. However, efforts are still required to reduce the barriers for virtual clinic implementation.

## Abbreviations

ICD-10-CM: International Classification of Diseases, Tenth Revision, Clinical Modification
NHI: National Health Insurance
NTUH: National Taiwan University Hospital
